# FLS2-BAK1 Extracellular Domain Interaction Sites Required for Defense Signaling Activation

**DOI:** 10.1371/journal.pone.0111185

**Published:** 2014-10-30

**Authors:** Teresa Koller, Andrew F Bent

**Affiliations:** 1 Department of Plant Pathology, University of Wisconsin – Madison, Madison, Wisconsin, United States of America; University of South Florida College of Medicine, United States of America

## Abstract

Signaling initiation by receptor-like kinases (RLKs) at the plasma membrane of plant cells often requires regulatory leucine-rich repeat (LRR) RLK proteins such as SERK or BIR proteins. The present work examined how the microbe-associated molecular pattern (MAMP) receptor FLS2 builds signaling complexes with BAK1 (SERK3). We first, using *in vivo* methods that validate separate findings by others, demonstrated that flg22 (flagellin epitope) ligand-initiated FLS2-BAK1 extracellular domain interactions can proceed independent of intracellular domain interactions. We then explored a candidate SERK protein interaction site in the extracellular domains (ectodomains; ECDs) of the significantly different receptors FLS2, EFR (MAMP receptors), PEPR1 (damage-associated molecular pattern (DAMP) receptor), and BRI1 (hormone receptor). Repeat conservation mapping revealed a cluster of conserved solvent-exposed residues near the C-terminus of models of the folded LRR domains. However, site-directed mutagenesis of this conserved site in FLS2 did not impair FLS2-BAK1 ECD interactions, and mutations in the analogous site of EFR caused receptor maturation defects. Hence this conserved LRR C-terminal region apparently has functions other than mediating interactions with BAK1. *In vivo* tests of the subsequently published FLS2-flg22-BAK1 ECD co-crystal structure were then performed to functionally evaluate some of the unexpected configurations predicted by that crystal structure. In support of the crystal structure data, FLS2-BAK1 ECD interactions were no longer detected in *in vivo* co-immunoprecipitation experiments after site-directed mutagenesis of the FLS2 BAK1-interaction residues S554, Q530, Q627 or N674. In contrast, *in vivo* FLS2-mediated signaling persisted and was only minimally reduced, suggesting residual FLS2-BAK1 interaction and the limited sensitivity of co-immunoprecipitation data relative to *in vivo* assays for signaling outputs. However, Arabidopsis plants expressing FLS2 with the Q530A+Q627A double mutation were impaired both in detectable interaction with BAK1 and in FLS2-mediated responses, lending overall support to current models of FLS2 structure and function.

## Introduction

Plants use pattern-recognition receptors (PRRs) as a first layer of defense against pathogens [1], [2]. In order to engineer plants with improved pathogen recognition abilities, it is important to understand the molecular details underlying the interaction of PRRs not only with their ligands but also with their co-receptors, immediate downstream targets and other partner proteins that facilitate appropriate signaling. Several PRRs have been identified in different plant species [reviewed in 1,2]. PRRs are localized at the plasma membrane where they monitor the apoplastic space for microbe-associated molecular patterns (MAMPs), damage-associated molecular patterns (DAMPs) and apoplastic effectors. Most known PRRs are receptor-like kinases (RLKs) or receptor-like proteins (RLPs). Both receptor types consist of an extracellular domain for ligand perception and a transmembrane domain, but only the RLKs have an intracellular kinase domain. Two of the best characterized PRRs, FLS2 and EFR [3], [4], carry large extracellular domains (ECDs, ectodomains) that predominantly consist of a leucine-rich repeat (LRR) domain [5], [6]. The genomes of Arabidopsis and other plants each encode hundreds of LRR receptor-like kinases (LRR-RLKs) with 4 to 28 repeat units of the LRR [7].

Receptors typically exhibit high specificity for ligands with which they interact, but cells also contain co-receptors and regulatory proteins that function together with receptors and do not necessarily exhibit specificity for only a single type of ligand [8], [9]. These co-receptors and regulatory proteins can be important facilitators or suppressors of signaling activation. They also allow signaling crosstalk at the plasma membrane, helping to coordinate appropriate downstream signaling in the presence of diverse endogenous and exogenous extracellular ligands. Important examples of regulatory/co-receptor RLKs include the SERK family members [8], [10], BIR family members [11], [12] and SOBIR1 [13].

SERK proteins have been identified in many different plant species. In Arabidopsis the family consists of five members (SERK1, SERK2, SERK3/BAK1, SERK4 and SERK5). They all have five LRRs in their ectodomain, share high overall sequence similarity and have redundant functions to various degrees. SERK proteins (mainly SERK3, also known as BAK1) have been shown to be involved in plant immunity in Arabidopsis, tomato and rice, through interactions with the receptors FLS2, EFR, PEPR1, PEPR2, Xa21, Ve1 and Eix1 [14]–[19]. The BAK1 co-receptor also contributes to somatic embryogenesis [20], [21] and to plant development through interaction with the brassinolide hormone receptor BRI1 [22], [23]. Despite impressive progress, much remains unknown about how the SERK proteins participate in all these different cell signaling tasks, and about the spatial expression of SERK proteins [24]. Studies of the SERK proteins are impeded by the redundant functions among family members and by pleiotropic effects when multiple SERK proteins are knocked out. As an example, *bak1^−^* Arabidopsis plants only have partially disrupted FLS2 signaling outputs [14], [15], [25], [26]. A possible means of circumventing this problem of SERK functional redundancy, adopted in the present study, is to identify the specific SERK interaction site of a partner receptor and then mutate that site. If all SERKs interact with a specific receptor at similar amino acids, this approach should impair the interaction of the receptor with all SERK family members.

Recent X-ray crystallography studies provided detailed insight into the interaction of the ectodomain of BAK1 with the ectodomains of FLS2 and BRI1 [27], [28], and the interaction of the ectodomains of SERK1 and BRI1 [29]. In all three cases the respective ligand promotes interaction between the ectodomains of the main receptors (FLS2 and BRI1) and the SERK co-receptors (BAK1 and SERK1). The ligand binds to the LRR domain of the main receptor, but the LRR domain of the SERK co-receptor also has multiple direct contacts with the ligand. It is surprising to see these fine-tuned co-receptor/ligand interactions, considering how many different known and potential unknown receptors and ligands BAK1 and SERK1 are able to interact with. Similar residues of the BAK1 and SERK1 ectodomains are involved in their interactions with FLS2 and BRI1. However, the residues on FLS2 and BRI1 ectodomains predicted to be used for the interactions with their SERK co-receptors are very different, not only in sequence but also in their location within the receptor LRR domain [27]–[29]. In BRI1 the residues interacting with co-receptors are located at the island domain, the last LRR, and the juxtamembrane domain, all close to the transmembrane domain. However, in FLS2 the BAK1-interacting residues in the crystal structure are located 108–300 amino acids from the predicted transmembrane domain, at repeats #18 to 26 of the LRR domain. This predicts a relatively recumbent orientation for the FLS2 ectodomain, bent down toward the plasma membrane (see Figure S3A).

FLS2 mediates perception of bacterial flagellin protein, an abundant MAMP, and FLS2 recognizes in particular a ∼20 amino acid region that is relatively conserved across flagellins from diverse Gram-negative bacteria [1], [30]. Many aspects of FLS2 structure and function have been characterized [reviewed in 31]. There is a third surprising feature of the FLS2-flg22-BAK1 ECD co-crystal structure [27]. Most research regarding FLS2 utilizes as ligand, in place of flagellin protein, a 22 amino acid “flg22” peptide whose sequence matches the recognized domain of *Pseudomonas aeruginosa* flagellin, or utilizes other small peptides based on similar sequences from various bacteria [30], [32], [33]. The FLS2-flg22-BAK1 ECD co-crystal structure predicts a tight pocket for the flg22 peptide, which may not be compatible with (allow sufficient space or sufficient ligand flexibility for) analogous binding of flg22 domains embedded within full-length flagellin proteins (discussed below).

In this study we first explored the possibility that a relatively universal SERK interaction site has evolved in the LRR domains of different SERK-interacting LRR-RLKs. We also showed that flg22-dependent FLS2 interaction with BAK1 occurs via the FLS2 extracellular domains – a result subsequently shown by alternative methods by Sun *et al.* (2103). We then performed site-directed mutagenesis and functional testing of predicted LRR-RLK receptor/SERK co-receptor interaction residues, and obtained *in vivo* evidence that supports models suggested by the recently published receptor/co-receptor co-crystal structures of truncated FLS2 and BAK1. The overall goal of this study was to furnish a more clear understanding of the requirements for formation of a signaling-competent plant basal immune system MAMP receptor – an understanding that may be essential to allow future engineering of PRRs with broadened or otherwise improved performance.

## Methods

### Arabidopsis and *Nicotiana benthamiana* transformation

The floral dip method was used to stably transform Arabidopsis *fls2^−^* and *efr^−^* plants. T1 seedlings were selected on 0.5x MS plates containing 25 mg/L kanamycin and 25 mg/L hygromycin. Leaves of 4-week-old *Nicotiana benthamiana* plants were infiltrated with *Agrobacterium tumefaciens* GV3101 containing the binary plasmids [34]. Proteins were harvested two days after *Agrobacterium tumefaciens* infiltration.

### Co-immunoprecipitation

Transiently transformed leaf tissue from *Nicotiana benthamiana* was infiltrated with 1 µM flg22 or 1 µM elf18, or with water for mock infiltration. After 2 minutes the leaf tissues were blotted dry and frozen in liquid N_2_. Then 200 mg of tissues were ground in 200 µl protein extraction buffer (50 mM Tris pH 7.5, 150 mM NaCl, 0.5% Triton X-100, 1x plant protease inhibitor cocktail (Sigma-Aldrich)). After centrifugation 300 µl supernatant was incubated with 3 µl 9E10 anti-myc antibody (Sigma-Aldrich or Covance) and rotated at 4°C for 1 h. 50 µl Protein A (Thermo Scientific) was added and the tubes were rotated at 4°C for an additional 2 h. After 3x washing with protein extraction buffer and 1x washing with ddH_2_O the beads were resuspended in 60 µl loading buffer and boiled at 95°C for 5 min. After centrifugation the supernatant was separated on two 8% SDS-PAGE gels. For protein detection the antibodies anti-HA-HRP, anti-myc rabbit and goat-anti-rabbit-HRP (Sigma-Aldrich) were used.

### Conservation mapping

Mapping of conserved regions of predicted LRR surfaces was performed using the Repeat Conservation Mapping (RCM) program at www.plantpath.wisc.edu/RCM [35], with heat map coloration range set to the minimal and maximal conservation scores of the data within each figure. The LRR domain sequences were obtained from The Arabidopsis Information Resource (TAIR) website at www.arabidopsis.org and from the National Center for Biotechnology Information (NCBI) website at www.ncbi.nlm.nih.gov. The following FLS2 non-Brassicaceae sequences were used: *Populus trichocarpa* (XP_002305701.1); *Vitis vinifer*a (XP_002272319.2); *Glycine max* (XP_003532650.1); *Lotus japonicus* (AER60531.1); *Ricinus communis* (XP_002519723.1); *Sorghum bicolor* (XP_002448543.1); *Oryza sativa Japonica* (CAE02151.2); *Oryza sativa Indica* (CAH68341.1); *Hordeum vulgare* (BAJ89141.1); *Brachypodium distachyon* (XP_003581675.1).

### Site-directed mutagenesis

Point mutations were generated according to the QuikChange mutagenesis kit (Agilent Technologies) on pENTR plasmids (Invitrogen) containing FLS2, FLS2-NoKinase or EFR with 35 S or native promoters [36]. Gateway LR Clonase II (Invitrogen) was used to transfer the construct into the binary plasmids pGWB13 or pGWB14 [37].

### EndoH assay

Leaf tissues (60 mg) from Arabidopsis T1 plants or from transiently transformed *Nicotiana benthamiana* plants were ground in 2x SDS buffer and boiled for 5 min at 95°C. After centrifugation for 10 min at 14000 rpm at 4°C supernatants were digested with Endoglycosidase H (New England BioLabs) as per manufacturer’s suggestion and separated on 8% SDS-PAGE gel. Proteins were detected using anti-HA-HRP antibody (Sigma-Aldrich).

### Seedling growth inhibition

T1 Arabidopsis seedlings were grown for 6 days on 0.5x MS plates with 25 mg/L kanamycin and 25 mg/L hygromycin and 200 mg/L cefotaxime. 24 seedlings per genotype, representing 24 independent transformation events, were transferred to 24-well-plates containing 1 ml 0.5x MS liquid media per well. 12 seedlings per genotype were grown for 14 days in wells containing 1 µM flg22 and 12 seedlings per genotype were grown for 14 days in wells containing only 0.5 x MS. Seedlings were then blotted dry and weighed. The weight of each flg22-treated seedling was divided by the average weight of the mock treated seedlings of the same genotype from the same experiment, prior to determination of experiment means and standard errors.

### Oxidative burst

Seven leaf discs were taken from six-week-old T1 Arabidopsis plants and incubated overnight in 1% DMSO solution. Peptide solution was added to the leaf discs and luminescence was measured by a plate reader for 0–30 min after addition of flg22 peptide. For measurement each leaf disc was in 100 µl peptide solution containing 0.5 µl 2 mg/ml horseradish peroxidase, 0.5 µl 2 mg/ml luminol in DMSO and 1 µM flg22.

## Results and Discussion

### Extracellular domain of FLS2 can mediate interaction with BAK1 in the presence of flg22

**Figure 1:**
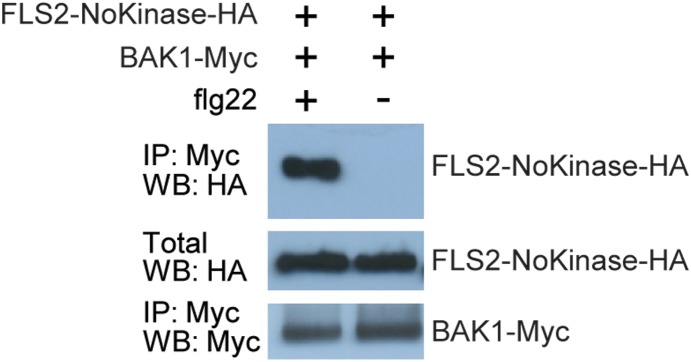
Extracellular domain of FLS2 can mediate interaction with BAK1. Co-immunoprecipitation experiments performed using *35S–FLS2-NoKinase-HA* (construct lacking the FLS2 intracellular domain) and *35S–BAK1-Myc* transiently expressed in *Nicotiana benthamiana* leaves by agroinfiltration. Samples were prepared for SDS-PAGE two days after agroinfiltration, two minutes after flg22 or water (mock) was infiltrated into leaves. IP: antibody used for immunoprecipitation prior to SDS-PAGE; WB: antibody used for immunodetection on protein blot; crude: SDS-PAGE and blotting of total (crude extract) protein samples. The experiment was repeated three times with similar results.

Full-length FLS2 and BAK1 do not detectably interact until exposure to flg22 or similar flagellin ligands, at which time interaction is immediately observed [14], [15], [38]. Flg22-elicited immune signaling then requires phosphorylation events among the respective kinase domains [26], [38], [39]. We hypothesized that the FLS2-BAK1 interaction is mediated not only intracellularly by the respective kinase domains, but also by interaction of the ectodomains. To test this we used a truncated FLS2 carrying the N-terminal ∼70% of the protein including the LRR and transmembrane domains but not the predicted intracellular domains (*FLS2-NoKinase-HA*; [36]). *FLS2-NoKinase-HA* was expressed in *Nicotiana benthamiana* together with a plasmid encoding a full-length, epitope tagged *BAK1-Myc*. The transiently transformed leaves were treated with flg22 and co-immunoprecipitation experiments were performed. BAK1 and FLS2-NoKinase interact in the presence of flg22, indicating that the kinase domain of FLS2 is not needed for interaction with BAK1 *in planta* (Figure 1).

Sun *et al.* 2013 also showed ECD mediation of FLS2-BAK1 interaction [27]. Their work utilized *in vitro* mixing experiments with purified recombinant proteins, or mutated BAK1 expressed in Arabidopsis protoplasts. Our results with mutated FLS2, tested in transgenic whole plants with *FLS2* expressed under control of *FLS2* promoter sequences, are complimentary and in agreement with the results of Sun *et al.* (2103), and reveal that intracellular/kinase domain interactions of these proteins are not required for flg22-stimulated FLS2-BAK1 interaction. It is also interesting to note the previously published finding that FLS2-FLS2 interaction occurs *in planta*, with either full-length FLS2 or FLS2-NoKinase constructs [36]. At least some FLS2 exists *in planta* in FLS2-FLS2 complexes, prior to and after flagellin or flg22 exposure. FLS2-BAK1 interaction after exposure to flg22 did not appreciably deplete the overall presence of co-immunoprecipitable FLS2-FLS2 complexes [36]. Hence findings that FLS2 and BAK1 interact via LRR domains suggest either that FLS2-FLS2 interactions utilize a different side or face of the FLS2 LRR than the region that interacts with BAK1, or that different sub-pools of FLS2 are at any given moment interacting with FLS2 or BAK1. The results of Albert *et al.* (2013) and Cao *et al.* (2013) are also relevant to these updated models of PRR receptor - co-receptor structure/function [39], [40]. Those studies demonstrated that *in planta* responses to flg22 are retained when hybrid FLS2 and BAK1 proteins are expressed in which the kinase domains of FLS2 and BAK1 have been reciprocally swapped [40], and that flg22-mediated FLS2-BIK1 disassociation and FLS2-BAK1 association still occur when FLS2 kinase domain mutations are present that block defense signaling. Schulze *et al.* 2010 and Schwessinger *et al.* 2011 showed that kinase-dead BAK1 still interact with FLS2, but impair FLS2 signaling [26], [38]. The evidence increasingly indicates that interactions of the FLS2 and BAK1 extracellular domains are a first step in flg22 perception that can proceed relatively independent of intracellular domain structural or functional interactions.

### Identification of a conserved region in the C-terminal LRRs of BAK1-interacting receptors

**Figure 2:**
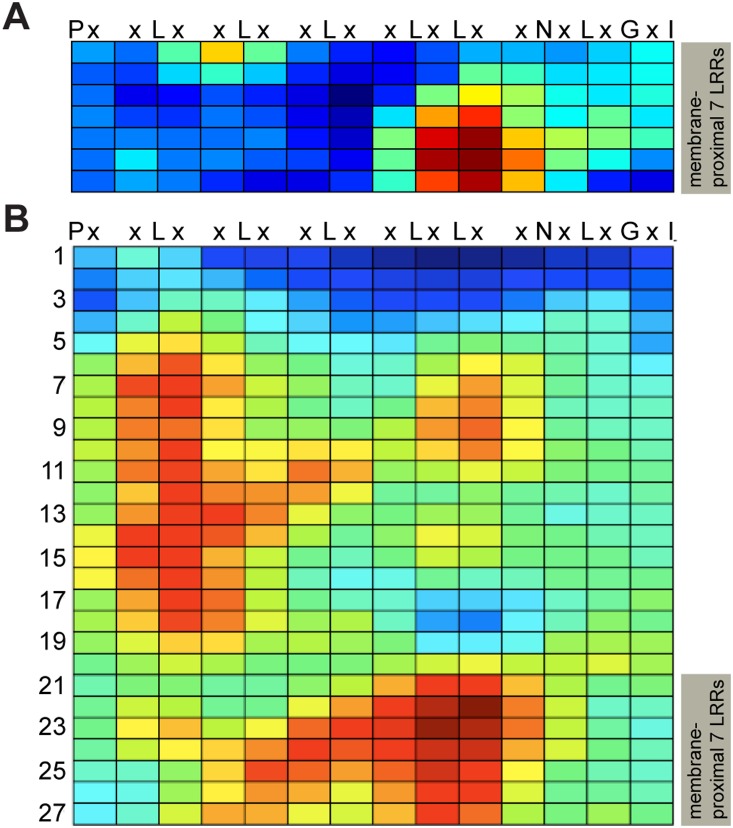
Repeat Conservation Mapping reveals conserved region near C-terminus of LRR domains of FLS2, EFR, BRI1 and PEPR1. Each row represents one leucine-rich repeat (LRR) and each square represents one solvent-exposed “x” amino acid position (as per LRR consensus sequence shown at the top). Conservation score at each amino acid position is center-weighted score for the cluster of 15, 20 or 25 predicted solvent-exposed LRR amino acids surrounding that site; blue: least conserved, red: most conserved. For FLS2, the seven rows of (A) are the same repeats (same residues) as rows 21–27 of (B). (**A**) Conservation map generated by comparing the most C-terminal seven repeats of the LRR sequences of the BAK1 interacting proteins FLS2, EFR, BRI1 and PEPR1. (**B**) Conservation map generated by comparing the entire FLS2 LRR domain sequences from eleven non-Brassicaceae plant species.

The SERK family members have been shown to interact with several different transmembrane LRR-RLKs involved in plant immunity and development. It is not known if the SERK interaction sites of these receptors evolved independently or originate from a common and potentially conserved SERK interaction site. We hypothesized the latter and also hypothesized that, to facilitate spatial proximity of potentially interacting extracellular domains, the relatively small ectodomains of SERK proteins would interact near the C-terminal end of the large LRR ectodomains of those partner receptors. Using Repeat Conservation Mapping [35] we searched the last seven repeats of the LRRs of the known Arabidopsis BAK1-interacting proteins FLS2 (28 total repeats in the LRR domain), EFR (21 LRRs), BRI1 (25 LRRs) and PEPR1 (26 LRRs), looking for the patch of solvent-exposed amino acids in this region that is most conserved across the four proteins. A conserved region of interest was identified (Figure 2A). Separately, we compared the solvent exposed amino acids of the whole LRR domains of eleven non-Brassicaceae FLS2s (Figure 2B). Both conservation maps revealed a conserved region at a similar location in the C-terminal LRRs. We hypothesized that this may be a somewhat universal site for interaction with SERK proteins.

### No disruption of FLS2-BAK1 interaction by mutations in the FLS2 LRR domain C-terminal region conserved among EFR, PEPR1, BRI1 and multiple FLS2s

**Figure 3:**
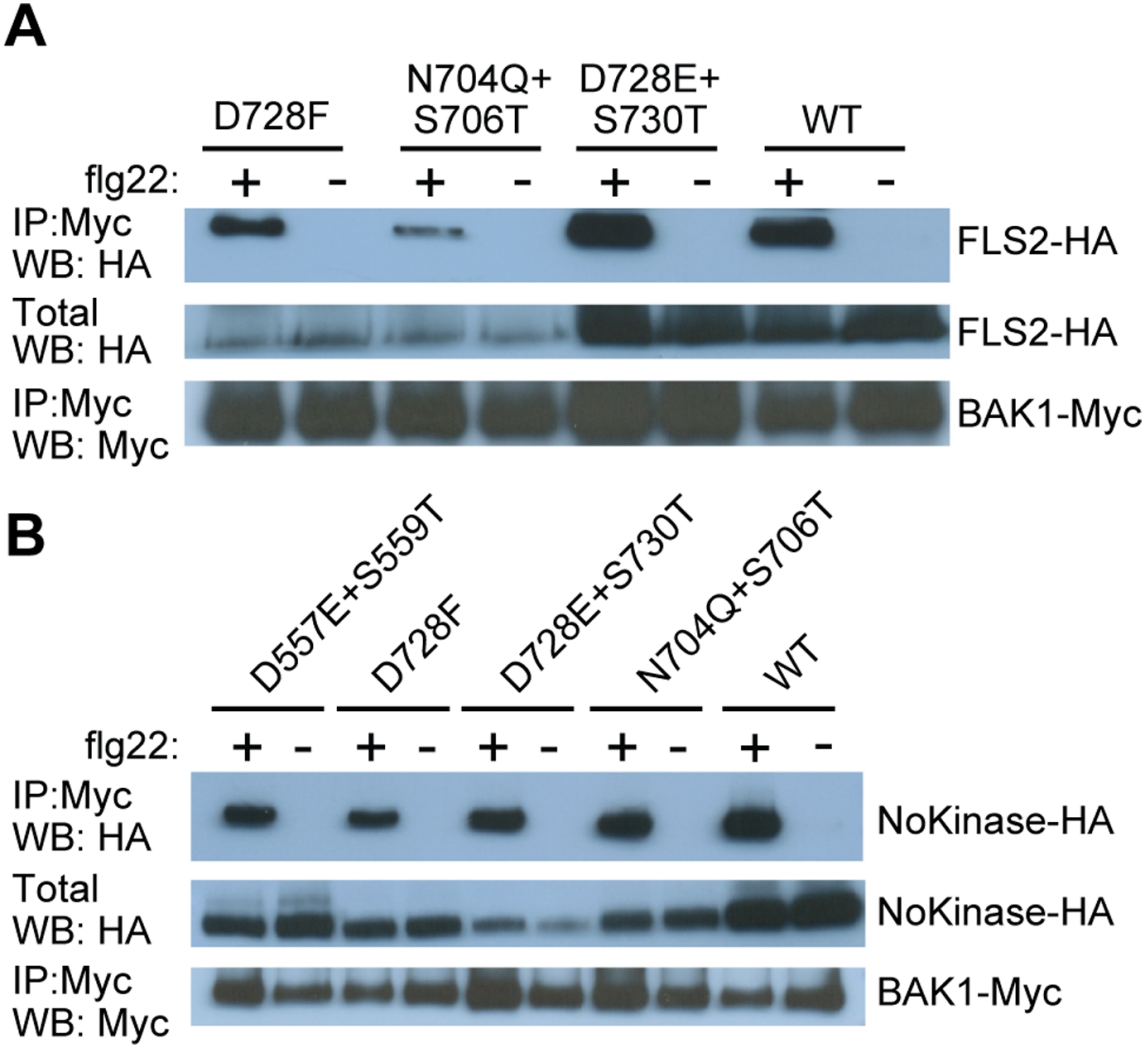
Mutations in the conserved C-terminal region of the FLS2 LRR domain did not have an impact on BAK1-FLS2 or BAK1-FLS2-NoKinase interaction. (**A**) Co-immunoprecipitation experiments performed using full-length *P_FLS2_-FLS2-HA*, with mutations as indicated or WT (no mutations), and *35S–BAK1-Myc*. (**B**) Co-immunoprecipitation experiments performed using *35S–FLS2-NoKinase-HA*, with mutations as indicated or WT (no mutations), and *35S–BAK1-Myc*. All samples in (A) and (B) are from *Nicotiana benthamiana.* Labeling as in Figure 1.

Site-directed mutagenesis was carried out to alter residues in the identified conserved LRR C-terminal region of FLS2 and EFR (Figure 2; Figure S1A–E). D557 and S559 mutations in FLS2 were included as control mutations located in LRR sites analogous to N704/S706 and D728/S730, but outside of the conserved LRR C-terminus. The amino acids were replaced with similar yet bulkier residues in order to impair interactions. The resulting full-length receptors were expressed in *N. benthamiana* and co-immunoprecipitation experiments were then carried out, using BAK1-Myc for pull-down in the presence and absence of the corresponding ligands flg22 and elf18 in the case of FLS2 or EFR, respectively. The mutations in FLS2 did not abolish the interaction with BAK1 in the presence of flg22 (Figure 3A). As is common for agroinfiltration experiments, variable levels of expression were observed for any single transgene-encoded FLS2 protein across replicates within or between experiments, but none of the mutant proteins was reproducibly present at levels different from transgene-encoded wild-type FLS2. To ensure that interaction of the kinase domains of FLS2 and BAK1 was not masking non-interaction of mutated FLS2 and BAK1 ectodomains, the same mutations were also placed into FLS2-NoKinase constructs. In these FLS2-NoKinase variants the mutations again did not prevent interaction with BAK1 (Figure 3B).

### Mutations in the conserved C-terminal LRR region of EFR cause EFR glycosylation/maturation defects

**Figure 4:**
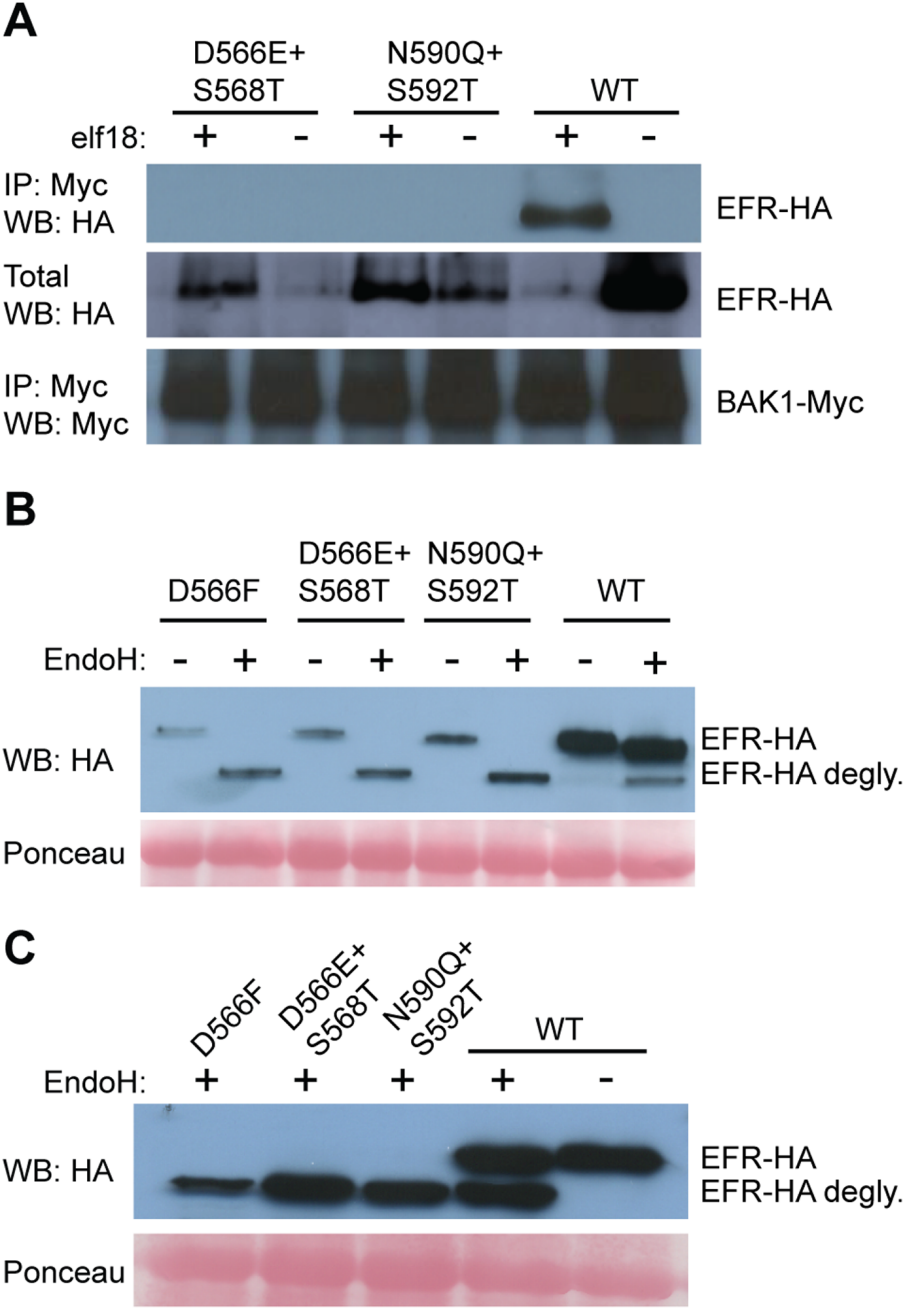
Mutations in the conserved C-terminal region of the EFR LRR domain disrupt EFR glycosylation and interaction with BAK1 in the presence of elf18. (**A**) Co-immunoprecipitation experiments performed using *P_EFR_-EFR-HA* with mutations as indicated or WT (no mutations), and *35S–BAK1-Myc*, in *Nicotiana benthamiana.* (**B, C**) Protein extracts from plants expressing *P_EFR_-EFR-HA* with mutations as indicated, or WT (no mutations), not digested or digested with endoglycosidase H (EndoH). Samples in (B) are from *Nicotiana benthamiana*, samples in (C) are from stably transformed *efr ^−^*Arabidopsis leaves. EndoH-resistant (mature) EFR is present in the EndoH-treated EFR wild type (WT) samples but is not detected for EFRs carrying the indicated mutations. Degly.: EFR pool deglycosylated by EndoH. Labeling as in Figure 1. Ponceau: blots treated with Ponceau stain to confirm even loading of total protein.

Mutations analogous to those of the preceding section were also engineered into *EFR*. These LRR domain C-terminal region mutations (Figure 2; Figure S1C, D) did cause disruption of interaction with BAK1 in the presence of elf18 (Figure 4A). This *in vivo* result could be attributable to direct impacts of the mutations on EFR-BAK1 interaction, or to defects in maturation and delivery of newly synthesized EFR out of the endoplasmic reticulum (ER) and golgi. Endoglycosidase H (EndoH) analyses were therefore conducted. EndoH cleaves incomplete glycosylation modifications present on proteins that have not successfully passed through the ER and related endomembrane systems [41], [42]. On the other hand, mature glycosylated proteins that are delivered to their functional location typically carry EndoH-resistant glycosylation [41], [42]. Treatment of the EFR protein extracts with EndoH revealed defects in the mutated EFR proteins, both in *N. benthamiana* and in stable transgenic Arabidopsis *efr-* plants expressing transgene *EFR* constructs driven by native *EFR* promoter sequences (Figure 4B, C). The mutations we generated in FLS2 full-length and FLS2-NoKinase did not result in glycosylation defects (Figure S2A, B). Häweker *et al.* 2010 [42] and Sun at al. 2012 [36] showed that single amino acid changes in glycosylation sites in the EFR ectodomain result in protein degradation and several studies reported the importance of intact glycosylation enzymes for successful processing and function of EFR [43]–[47]. FLS2 is less sensitive to mutations in glycosylation sites [36], [42]. The N590Q+S592T mutations that we placed in EFR are indeed in a Nx(S/T) predicted glycosylation site [48]. However, the EFR mutations D566E+S568T and D566F are not, yet they still disrupted correct EFR processing.

Taken together, the above results suggest that functional roles of the LRR C-terminal conserved domain of BAK1-interacting proteins (Figure 2) do not serve as a universal SERK protein interaction site. However, in EFR the integrity of this site is important for correct protein processing.

### FLS2 mutations in proposed FLS2-BAK1 ECD interaction residues disrupt FLS2-BAK1 interaction in the presence of flg22

While the above work was in progress the crystal structure of FLS2-flg22-BAK1 ECD became available [27]. That important work identified in detail the interaction sites of the FLS2 and BAK1 ectodomains. Because the data are for *in vitro* crystallized protein complexes of isolated LRR domains, they may or may not capture the most functionally prominent *in vivo* configurations. Sun *et al.* [27] therefore functionally tested BAK1 mutations, and also tested FLS2 mutations in repeats #9, 11, 14 and 15 of the LRR that are predicted to mediate interaction with flg22. The sites on FLS2 predicted to mediate interaction with BAK1 did not receive mutational testing. In order to test *in vivo* the significance of these FLS2 BAK1-interaction residues, which are likely to also mediate interaction of FLS2 with other SERK proteins, we performed site-directed mutagenesis on *FLS2-NoKinase* and full-length *FLS2*.

**Figure 5:**
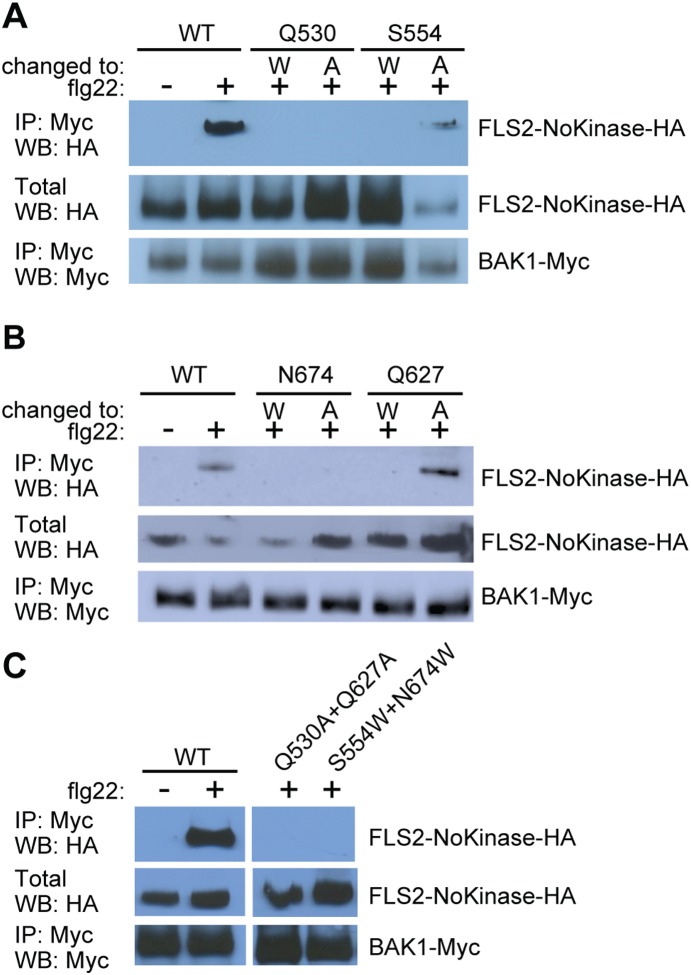
FLS2 residues Q530, S554, Q627 and N674 are important for FLS2-BAK1 ectodomain interaction in the presence of flg22. Co-immunoprecipitation experiments performed in *N. benthamiana* with 35S*–FLS2-NoKinase-HA* with mutations as indicated or WT (no mutations), and with *35S–BAK1-Myc*. Flg22-dependent interaction between FLS2-NoKinase and BAK1 not detected for (**A**) FLS2 carrying Q530A, Q530W or S554W mutations, (**B**) FLS2 carrying N674A, N674W or Q627W mutations, or (**C**) FLS2 carrying Q530A+Q627A or S554W+N674W double mutations. Labeling as in Figure 1.

For FLS2 amino acids predicted in the crystal structure to form FLS2-BAK1 interaction sites [27], we changed single residues to alanine (small and relatively inactive) or to tryptophan (bulky). In addition to the single mutations we made two *FLS2-NoKinase* constructs with double mutations and one full-length *FLS2* construct with a double mutation. We had previously shown that the FLS2-NoKinase used in this work performed similarly to FLS2-full-length in flg22-dependent BAK1 co-immunoprecipitation experiments (Figure 3A, B). In *in vivo* tests of the newly predicted FLS2-BAK1 interaction sites, mutation of FLS2 residues Q530, S554, Q627 or N674 to tryptophan disrupted the flg22-stimulated interaction of FLS2-NoKinase with BAK1 (Figure 5A, B). The interaction was disrupted as well when FLS2 Q530 and N674 were changed to alanine (Figure 5A, B). However, the FLS2 S554 and Q627 single mutations to alanine had much less impact on flg22-dependent interaction with BAK1 (Figure 5A, B), suggesting a stronger role for Q530 and N674 than S554 or Q627 in mediating FLS2-BAK1 interaction. The double alanine mutation Q530A+Q627A and the double tryptophan mutation S554W+Q627W disrupted BAK1 interaction as well (Figure 5C). The presence of abundant EndoH-insensitive bands suggested that FLS2 maturation had proceeded successfully for each of the representative FLS2 mutants S554A, Q627W and Q530A+Q627A (Figure S2C).

### Arabidopsis *fls2^−^* plants carrying FLS2-Q530A+Q627A have impaired FLS2-mediated signaling outputs

**Figure 6:**
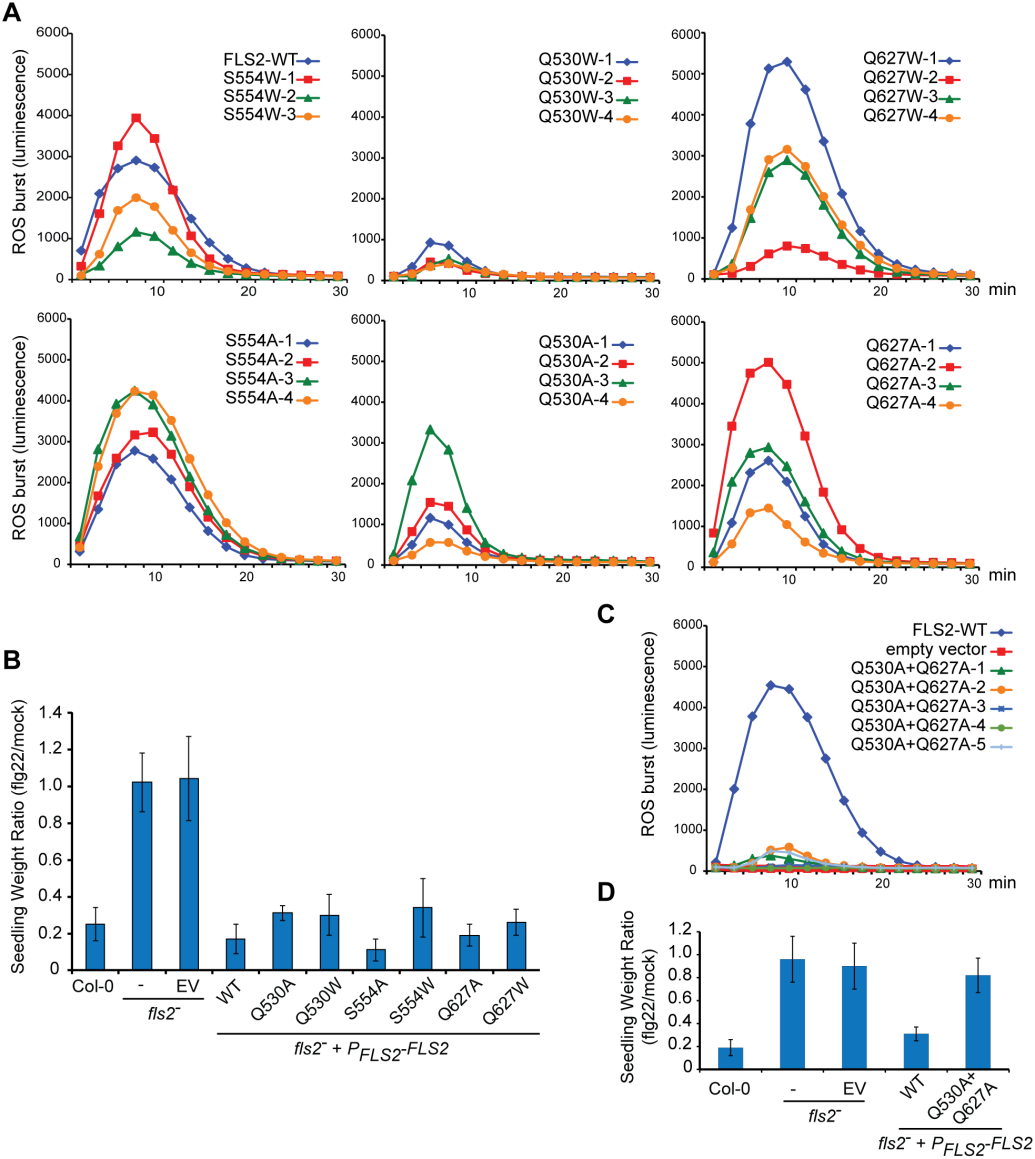
FLS2 signaling output impaired to various degrees in Arabidopsis *fls2^−^* plants expressing FLS2 mutations that impact FLS2-BAK1 interaction. (**A**) Reactive oxygen species (ROS) production in response to flg22 in Arabidopsis Col-0 *fls2^−^* plants stably transformed to express full-length FLS2 proteins carrying single mutations as noted, under control of *FLS2* promoter sequences. For each mutation, ROS production was recorded for 30 min. and the average for seven separately monitored leaf discs is shown for each of four independent transgenic lines (or three lines for S554W). WT: Average ROS response for six independent *fls2^−^* transformants expressing wild-type FLS2 (42 total leaf discs for WT), from same experiment. (**B**) FLS2-mediated seedling growth inhibition (SGI) in response to flg22, for plant lines as in (A). Mean and std. error of mean shown for six to eight independent transformants for each *FLS2* construct. (**C**) ROS experiment as in (A), except with five independent lines expressing FLS2 Q530A+Q627A double mutations. (**D**) Seedling growth experiment as in (B), except with twelve independent lines expressing Q530A+Q627A double mutations.

To investigate if mutations in predicted FLS2 BAK1-interaction residues not only disrupt FLS2-BAK1 interactions in co-immunoprecipitation experiments but also have an impact on FLS2 signaling, we made the analogous single mutations and one of the double mutations in full-length *FLS2s.* We then tested FLS2 signaling in stably transformed *fls2^−^* Arabidopsis plants containing the mutated and HA-tagged full-length *FLS2*s under control of native *FLS2* promoter sequences. The two most widely used assays for FLS2 signaling were utilized: ROS burst assays and seedling growth inhibition assays [1]. Surprisingly, *in vivo* FLS2-mediated signaling persisted and was only minimally reduced in plants expressing most single-mutant forms of FLS2 (Figure 6A, B), including mutants that exhibited no detectable flg22-induced co-immunoprecipitation with BAK1 (Figure 5A, B). As a general trend across the multiple independent transgenic lines tested for each *FLS2* construct, mutations to alanine allowed stronger FLS2 signaling than mutations to tryptophan (Figure 6A, B). The results suggest that reduced-affinity or more transient interactions of FLS2 and BAK1 occur with many of the FLS2 mutants described in Figures 5 and 6, and that those interactions are sufficient for flg22-stimulated FLS2 signaling even if the stability of FLS2-BAK1 interactions is reduced below levels detectable in standard co-immunoprecipitation experiments. Although some FLS2 signaling capacity was still conferred by FLS2 constructs mutated at single predicted FLS2 BAK1-interaction sites, with the double mutation Q530A+Q627A FLS2-mediated signaling was significantly impaired (Figure 6C, D), supporting current models of FLS2 structure and function.

Alternative hypotheses, other than reduced-affinity or more transient interactions of FLS2 and BAK1, can be formulated regarding the continued signaling by the FLS2 single mutants of Figure 5 and 6. For example, small sub-populations of FLS2 receptors (sufficient to initiate the levels of defense signaling observed in Figure 6) may exist in the cell that, because of different localization or post-translational modifications, continue to exhibit robust flg22-dependent interaction with BAK1 despite presence of mutations that disrupt interaction between most of the cellular FLS2 and BAK1. As another possibility, the single mutations that transition FLS2 away from high-affinity flg22-dependent binding with BAK1 may have allowed or even enhanced interaction with other SERK proteins, to an extent that allows defense signaling.

LRRs are a protein structure evolved to display widely varying surface amino acid combinations on a relatively invariant scaffold [5], [6]. A previous study of over 1200 FLS2 LRR mutations of predicted LRR solvent-exposed residues at and adjacent to flg22 binding sites, carrying changes to all possible amino acids (i.e., not just to alanine), found that the vast majority of LRR surface mutations do not disrupt FLS2 function [49]. Thus the structural alterations caused by the FLS2 mutations of the present study are likely to be highly local. Their disruption of FLS2-BAK1 interactions detected via co-immunoprecipitation supports the relevance of the FLS2-flg22-BAK1 configuration in the published co-crystal structure. Mutation of FLS2 residues D557 and S559, which reside close to but outside of the BAK1-interaction residues in the solved crystal structure ([27], Figure S1), did not disrupt flg22-stimulated FLS2-BAK1 co-immunoprecipitation (Figure 3). Hence the functional disruption of signaling caused by the presumably additive effect of two alanine substitutions in FLS2 Q530A+Q627A provides further *in vivo* functional evidence indicating the requirement for this site both for FLS2-BAK1 interaction and for flg22 induction of FLS2-dependent immune signaling. Our results also indicate that, if SERK proteins other than BAK1 make residual contributions to FLS2 activation (as is suggested above and in the literature [14], [15], [25], [26]), the FLS2 Q530A+Q627A mutations are sufficient to disrupt functional signaling mediated by those interactions as well.

### Closing Observations

In this study we explored the idea of a universal SERK protein interaction site in the C-terminal repeats of the LRR ectodomains of receptors known to interact with SERK proteins. However, mutagenesis of a possible BAK1 interaction site in the ectodomains of FLS2 and EFR did not confirm this hypothesis. The subsequently available FLS2-flg22-BAK1 and BRI1-brassinolide-SERK1 extracellular domain crystal structures [27] [29], and the mutational studies in the present work, instead suggest a fine-tuned interaction unique for each receptor/ligand/co-receptor complex. SERK1 and BAK1 use similar residues to interact with the BRI1 and FLS2 ectodomains, respectively. However, the SERK-interacting residues in the ectodomains of BRI1 and FLS2 are very different in terms of both the amino acid identities and their location along the large LRR macromolecule, and thus may have evolved separately.

The LRR surface region exhibiting conservation between FLS2, EFR, PEPR1 and BRI1 (Figure 2A) spans four repeats of the LRR, but overlaps with the larger region highlighted in Figure 2B that is conserved across diverse FLS2 proteins and spans the final seven repeats of the LRR. Within the larger conserved region, the residues that are further to the left as shown in Figure 2B (or Figure S1E) encompass the BAK1 interaction site, but the residues on the right do not. The present study detected no impact of mutations in FLS2 in the Figure 2A conserved region, which in FLS2 is the same as the bottom right of the larger conserved region of Figure 2B. A previous study from our group [35] reported little or no functional impact of mutations in the upper-right area of the conserved region (the darkest red/most conserved area of Figure 2B). In that study, libraries of changes to all possible amino acids were made at the four FLS2 residues D605, S607, F633 and S634, directly above the N704/S706 and D728/S730 residues targeted in this study but in repeats #22 and 23 [35]. Hence it is intriguing that this right side of the region highlighted in Figure 2B, which lies along the concave β-strand surface of repeats #21–27, is highly tolerant of mutations despite being relatively conserved across FLS2 proteins from diverse plant species. It remains of interest to discover the function of this portion of the FLS2 LRR.

As a separate but related matter, it is intriguing that the set of BAK1-interacting residues of FLS2 lie not only within regions highly conserved across FLS2 proteins from diverse plant species (e.g., Q627 and N674, Figure S1E), as might be expected, but also outside of conserved regions (e.g., Q530 and S554, Figure S1E). Figure 2B and Figure S1E show regions of LRR surface residue conservation in a comparison among FLS2s from non-Brassicaceae plant species (see Methods). But even in maps of conservation among FLS2s only from Brassicaceae species (see for example [35]), the region around Arabidopsis FLS2 residues Q627 and N674 is strongly conserved while Q530, S554 and adjacent BAK1-interacting residues [27] are in an LRR surface region that is less conserved. This raises the hypothesis that there is a functionally relevant diversification of SERKs and/or this upper portion of the SERK-interaction site of FLS2, even across Brassicaceae species.

The relevance of the FLS2-flg22-BAK1 co-crystal structure to actual configurations of the protein complex within plant cells would gain stronger support if more features of the crystal structure were reconciled with other findings regarding plant FLS2s and flagellin detection. We noted in the Introduction the concern that the co-crystal, made with flg22 peptide, may not allow enough space for docking of a full-length flagellin protein at the appropriate location. Figure S3 shows hypothetical alignments of the FLS2-flg22-BAK1 ECD structure (PDB ID: 4MN8) with the structure of one Salmonella flagellin protein (PDB ID: 3A5X), placing the flg22 region of 3A5X near the apparent flg22 binding sites of FLS2 and BAK1 while attempting to minimize co-occupancy of the same space by two different molecules. The FLS2 LRR, which is notably lacking in ‘loop-out’ or non-LRR-consensus regions, is likely to be relatively inflexible. Flagellin monomers in solution (not polymerized with other flagellins to form flagella-like structures) are likely to be more flexible than shown, particularly in the region of the flg22 residues that form a less ordered linker between two alpha-helical regions [50], [51] (see Figure S3F). Nevertheless, space-filling models (e.g., Figure S3D) demonstrate the difficulty of docking a large flagellin onto the requisite FLS2 LRR sites while also allowing space for BAK1 and not allowing co-occupancy of identical space. Importantly, even in hypothesized configurations (not shown) that might allow space for a more flexible full-length flagellin to interact with FLS2 and BAK1, the flg22 residues within a flagellin protein are apparently constrained in ways that would restrict simultaneous interaction with the majority of the FLS2 LRR surface residues that interact with the elongated flg22 in the published FLS2-flg22-BAK1 co-crystal structure (e.g., Figure S3E). FLS2, flagellins and BAK1 may associate *in vivo* in configurations that depart significantly from the co-crystal structure. However, numerous aspects of the published FLS2-flg22-BAK1 co-crystal structure are substantiated by experimental evidence ([27]; references therein; present study). Hence we consider it equally likely that the published FLS2-flg22-BAK1 co-crystal is essentially correct in representing *in vivo* configurations, and predict that flagellin proteins within plants must be fragmented rather than intact in order to form the FLS2-flagellin-BAK1 complexes that elicit plant innate immune system activation.

In the future, it also will be interesting to compare more receptor/ligand/co-receptor signaling complexes in order to learn more about the functional plasticity of co-receptors. As one example, a ligand-mediated EFR-BAK1 ectodomain complex is likely to initiate EFR signaling. Interestingly, when EFR from Arabidopsis was transferred to *Nicotiana benthamiana* or tomato (which lack an endogenous EFR) it triggered an elf18-activated immune response, indicating functional interaction of AtEFR with SERK proteins from *Nicotiana benthamiana* and tomato [52]. Thus one or more SERK proteins apparently carry sufficient structure-function plasticity to interact with different receptors even from diverse plant species, while complying with the fine-tuned sequence constraints of the resulting receptor/ligand/co-receptor complexes. For future engineering of PRR receptors with novel ligand specificities it will be important to ensure presence of an intact SERK protein interaction site in the ectodomain of the PRR, close to or overlapping with the ligand binding site, and ensure that the co-receptors also can form PRR/ligand/co-receptor complexes with the novel ligands for which new recognition specificity is sought.

## Supporting Information

Figure S1 **Mutation sites in FLS2 and EFR ECDs. (A)** Sites subjected to site-directed mutagenesis in the FLS2 LRR domain. Only repeats 17–28 are shown (FLS2: total 28 repeats). Green: mutation sites in the conserved LRR domain C-terminus (see also (B, D, E)). Blue: “control” mutations; sites similar to N704/D728 and S706/S730 but outside of the conserved region; blue “control” sites also adjacent to but outside of FLS2 BAK1-interaction site. Orange: mutation sites based on FLS2 BAK1-interaction sites in the FLS2-flg22-BAK1 ECD co-crystal structure. **(B)** Mutation sites as described in (A), using same color scheme as in (A). Structure is PDB ID: 4MN8 with FLS2 backbone as black ribbon, BAK1 backbone as light blue ribbon, and flg22 backbone as red ribbon. Space-filling spheres show side-chains only for mutagenized sites. **(C)** Mutation sites in the EFR LRR domain. Only repeats 17–21 are shown (EFR: total 21 repeats). Green: mutation sites in the conserved LRR domain C-terminus (see also (D)). **(D, E)** Regional LRR surface conservation maps from Arabidopsis FLS2, EFR, PEPR1 and BRI1 (D) or eleven non-Brassicaceae FLS2s (E), as shown and described in Figure 2, with x’s at the FLS2 and EFR LRR domain amino acid positions described above that were subjected to site-directed mutagenesis in the present study.
(PDF)

Figure S2 **EndoH assay reveals no glycosylation defects in mutated FLS2 and FLS2-NoKinase. (A)** Protein extracts from Arabidopsis *fls2^−^* leaves carrying *P_FLS2_-FLS2-HA* (with mutations as indicated, or WT = no mutations), not digested (−) or digested (+) with endoglycosidaseH (EndoH). An EndoH-resistant protein pool (characteristic of mature glycosylated proteins) is visible in all EndoH-treated samples. **(B)** Protein extracts from *Nicotiana benthamiana* carrying *35S–FLS2-NoKinase-HA* (with mutations as indicated), digested with EndoH. An EndoH-resistant protein pool is visible in all EndoH-treated samples. Mutations D557E+S559T were included as control mutations located in sites of a single LRR repeat analogous to D728E+S730T, but outside of the conserved LRR C-terminus. **(C)** Protein extracts from Arabidopsis *fls2^−^* seedlings carrying *P_FLS2_-FLS2-HA* (with mutations as indicated, or WT = no mutations), digested with EndoH. An EndoH-resistant protein pool is visible in all EndoH-treated samples except for the empty vector (EV) control. Ponceau stained blot shows similar loading of total protein in all lanes including EV negative control. Degly.: FLS2 pools deglycosylated by EndoH.
(PDF)

Figure S3 **Hypothetical docking of full-length flagellin structure (PDB ID: 3A5X) to FLS2-flg22-BAK1 structure (PDB ID: 4MN8) illustrates minimal space for flagellin inside FLS2 LRR, and constraints to flg22 contact with FLS2 LRRs #3–15 if flg22 region is held within full-length flagellin.** **(A), (B), (C)** Flagellin, hypothetically positioned so that flg22 residues within full-length flagellin are near the flg22 binding sites of FLS2 and BAK1. PDB structures 3A5X and 4MN8 superimposed at same scale; (B) and (C) are 90^o^ rotated views of (A). Light blue: flagellin; red: flg22 residues within flagellin (3A5X). Dark blue: FLS2 LRR; green: BAK1 LRR; yellow: flg22 co-crystallized with FLS2 and BAK1 LRRs (4MN8). **(D)** Same view as (C), with space-filling representation of flagellin to more clearly illustrate impossible overlap of flagellin and FLS2 residues in same spatial locations in this arrangement (and other arrangements) of 3A5X and 4MN8. FLS2 and BAK1 side-chains omitted for clarity. **(E)** PyMol alignment of flg22 (yellow, in structure 4MN8) and flg22 region within flagellin (red, in structure 3A5X). Lower portions of flg22 in (E) (the yellow residues that are not proximal to red residues) are the N-terminal 7 residues of flg22 that associate with FLS2 LRRs #3–7 (FLS2 and BAK1 not shown, for clarity). **(F)** Full length flagellin (PDB structure 3A5X) colored as in (A) but shown by itself, showing that flg22 region forms a less-ordered hinge region between flanking pairs of alpha-helical bundles.
(PDF)
