# Supplementary material for: FLS2-BAK1 Extracellular Domain Interaction Sites Required for Defense Signaling Activation
Source: PLoS One. 2014 Oct 30;9(10):e111185. doi: 10.1371/journal.pone.0111185 (PMC4214723; doi:10.1371/journal.pone.0111185)
Supplement: Figure S1 — Mutation sites in FLS2 and EFR ECDs. (A) Sites subjected to site-directed mutagenesis in the FLS2 LRR domain. Only repeats 17–28 are shown (FLS2: total 28 repeats). Green: mutation sites in the conserved LRR domain C-terminus (see also (B, D, E)). Blue: “control” mutations; sites similar to N704/D728 and S706/S730 but outside of the conserved region; blue “control” sites also adjacent to but outside of FLS2 BAK1-interaction site. Orange: mutation sites based on FLS2 BAK1-interaction sites in the FLS2-flg22-BAK1 ECD co-crystal structure. (B) Mutation sites as described in (A), using same color scheme as in (A). Structure is PDB ID: 4MN8 with FLS2 backbone as black ribbon, BAK1 backbone as light blue ribbon, and flg22 backbone as red ribbon. Space-filling spheres show side-chains only for mutagenized sites. (C) Mutation sites in the EFR LRR domain. Only repeats 17–21 are shown (EFR: total 21 repeats). Green: mutation sites in the conserved LRR domain C-terminus (see also (D)). (D, E) Regional LRR surface conservation maps from Arabidopsis FLS2, EFR, PEPR1 and BRI1 (D) or eleven non-Brassicaceae FLS2s (E), as shown and described in Figure 2, with x’s at the FLS2 and EFR LRR domain amino acid positions described above that were subjected to site-directed mutagenesis in the present study. (PDF) [file pone.0111185.s001.pdf]

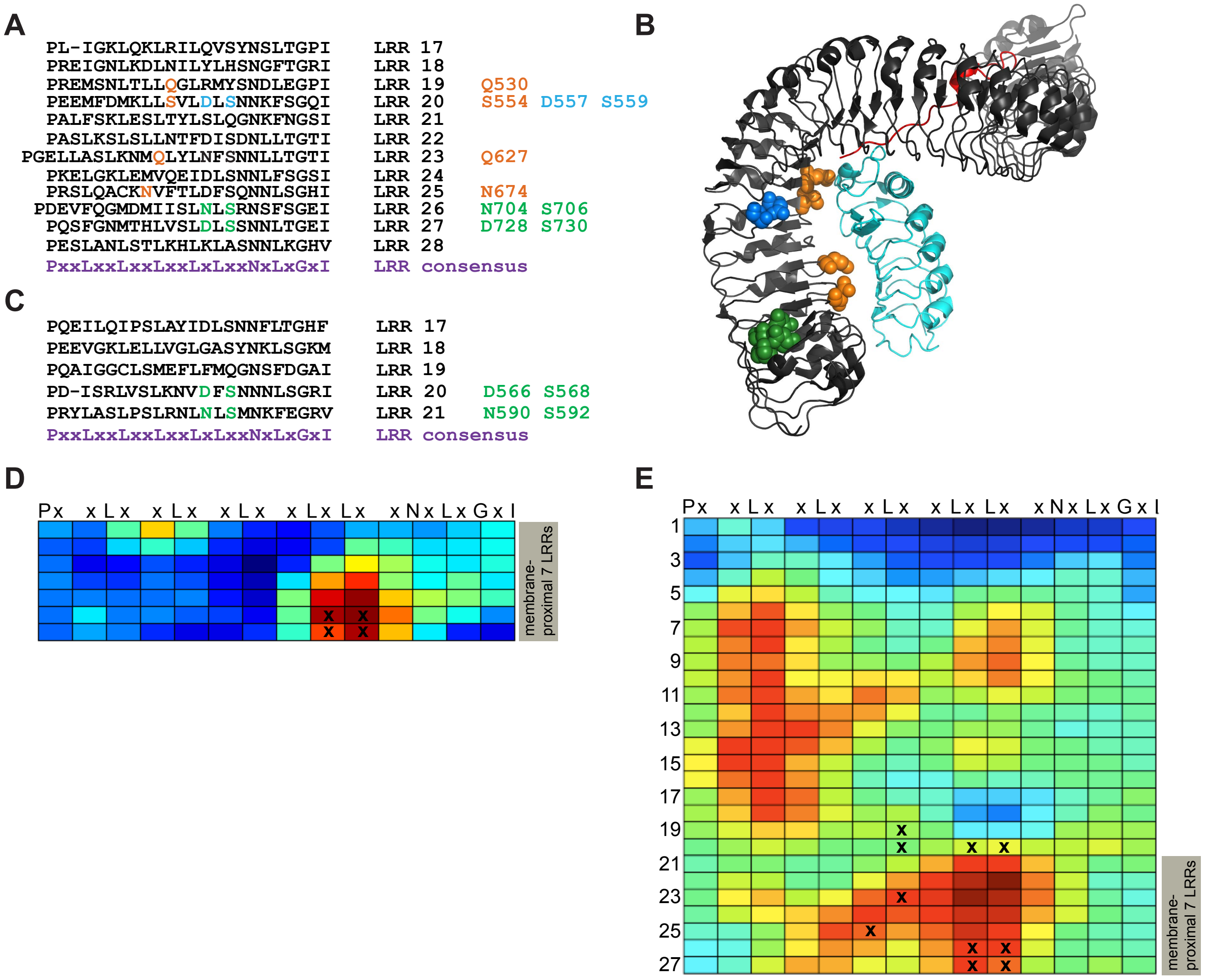

Supplemental Figure 1: Mutation sites in FLS2 and EFR ECDs. (A) Sites subjected to site-directed mutagenesis in the FLS2 LRR domain. Only repeats 17-28 are shown (FLS2: total 28 repeats). Green: mutation sites in the conserved LRR domain C-terminus (see also (B, D, E)). Blue: “control” mutations; sites similar to N704/D728 and S706/S730 but outside of the conserved region; blue “control” sites also adjacent to but outside of FLS2 BAK1-interaction site. Orange: mutation sites based on FLS2 BAK1-interaction sites in the FLS2-flg22-BAK1 ECD co-crystal structure. **(B)** Mutation sites as described in (A), using same color scheme as in (A). Structure is PDB ID: 4MN8 with FLS2 backbone as black ribbon, BAK1 backbone as light blue ribbon, and flg22 backbone as red ribbon. Space-filling spheres show side-chains only for mutagenized sites. **(C)** Mutation sites in the EFR LRR domain. Only repeats 17-21 are shown (EFR: total 21 repeats). Green: mutation sites in the conserved LRR domain C-terminus (see also (D))**. (D, E)** Regional LRR surface conservation maps from Arabidopsis FLS2, EFR, PEPR1 and BRI1 (D) or eleven non-Brassicaceae FLS2s (E), as shown and described in Figure 2, with x’s at the FLS2 and EFR LRR domain amino acid positions described above that were subjected to site-directed mutagenesis in the present study.
