# Supplementary material for: FLS2-BAK1 Extracellular Domain Interaction Sites Required for Defense Signaling Activation
Source: PLoS One. 2014 Oct 30;9(10):e111185. doi: 10.1371/journal.pone.0111185 (PMC4214723; doi:10.1371/journal.pone.0111185)
Supplement: Figure S2 — EndoH assay reveals no glycosylation defects in mutated FLS2 and FLS2-NoKinase. (A) Protein extracts from Arabidopsis fls2− leaves carrying PFLS2-FLS2-HA (with mutations as indicated, or WT = no mutations), not digested (−) or digested (+) with endoglycosidaseH (EndoH). An EndoH-resistant protein pool (characteristic of mature glycosylated proteins) is visible in all EndoH-treated samples. (B) Protein extracts from Nicotiana benthamiana carrying 35S–FLS2-NoKinase-HA (with mutations as indicated), digested with EndoH. An EndoH-resistant protein pool is visible in all EndoH-treated samples. Mutations D557E+S559T were included as control mutations located in sites of a single LRR repeat analogous to D728E+S730T, but outside of the conserved LRR C-terminus. (C) Protein extracts from Arabidopsis fls2− seedlings carrying PFLS2-FLS2-HA (with mutations as indicated, or WT = no mutations), digested with EndoH. An EndoH-resistant protein pool is visible in all EndoH-treated samples except for the empty vector (EV) control. Ponceau stained blot shows similar loading of total protein in all lanes including EV negative control. Degly.: FLS2 pools deglycosylated by EndoH. (PDF) [file pone.0111185.s002.pdf]

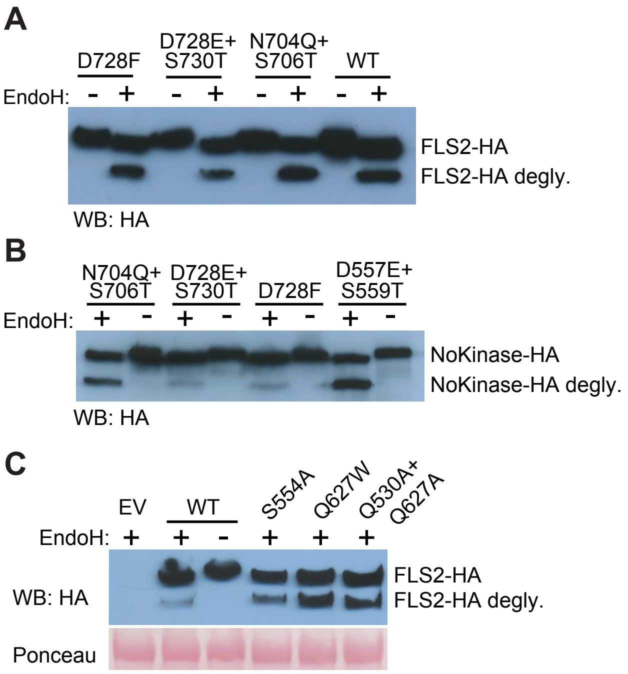

Supplemental Figure 2: EndoH assay reveals no glycosylation defects in mutated FLS2 and FLS2-NoKinase. (A) Protein extracts from Arabidopsis *fls2*⁻ leaves carrying P_{FLS2}-*FLS2-HA* with mutations as indicated or WT (no mutations), not digested (-) or digested (+) with endoglycosidaseH (EndoH). An EndoH-resistant protein pool (characteristic of mature glycosylated proteins) is visible in all EndoH-treated samples. **(B)** Protein extracts from *Nicotiana benthamiana* carrying *35S-FLS2-NoKinase-HA* with mutations as indicated, not digested (-) or digested (+) with endoglycosidaseH (EndoH). An EndoH-resistant protein pool is visible in all EndoH-treated samples. Mutations D557E+S559T were included as control mutations located in sites of a single LRR repeat analogous to D728E+S730T, but outside of the conserved LRR C-terminus. **(C)** Protein extracts from Arabidopsis *fls2*⁻ seedlings carrying P_{FLS2}-*FLS2-HA* with mutations as indicated or WT (no mutations), digested with EndoH. An EndoH-resistant protein pool is visible in all EndoH-treated samples except for the empty vector (EV) control. Ponceau stained blot shows similar loading of total protein in all lanes including EV negative control. Degly.: FLS2 pools deglycosylated by EndoH.
