# Supplementary material for: FLS2-BAK1 Extracellular Domain Interaction Sites Required for Defense Signaling Activation
Source: PLoS One. 2014 Oct 30;9(10):e111185. doi: 10.1371/journal.pone.0111185 (PMC4214723; doi:10.1371/journal.pone.0111185)
Supplement: Figure S3 — Hypothetical docking of full-length flagellin structure (PDB ID: 3A5X) to FLS2-flg22-BAK1 structure (PDB ID: 4MN8) illustrates minimal space for flagellin inside FLS2 LRR, and constraints to flg22 contact with FLS2 LRRs #3–15 if flg22 region is held within full-length flagellin. (A), (B), (C) Flagellin, hypothetically positioned so that flg22 residues within full-length flagellin are near the flg22 binding sites of FLS2 and BAK1. PDB structures 3A5X and 4MN8 superimposed at same scale; (B) and (C) are 90o rotated views of (A). Light blue: flagellin; red: flg22 residues within flagellin (3A5X). Dark blue: FLS2 LRR; green: BAK1 LRR; yellow: flg22 co-crystallized with FLS2 and BAK1 LRRs (4MN8). (D) Same view as (C), with space-filling representation of flagellin to more clearly illustrate impossible overlap of flagellin and FLS2 residues in same spatial locations in this arrangement (and other arrangements) of 3A5X and 4MN8. FLS2 and BAK1 side-chains omitted for clarity. (E) PyMol alignment of flg22 (yellow, in structure 4MN8) and flg22 region within flagellin (red, in structure 3A5X). Lower portions of flg22 in (E) (the yellow residues that are not proximal to red residues) are the N-terminal 7 residues of flg22 that associate with FLS2 LRRs #3–7 (FLS2 and BAK1 not shown, for clarity). (F) Full length flagellin (PDB structure 3A5X) colored as in (A) but shown by itself, showing that flg22 region forms a less-ordered hinge region between flanking pairs of alpha-helical bundles. (PDF) [file pone.0111185.s003.pdf]

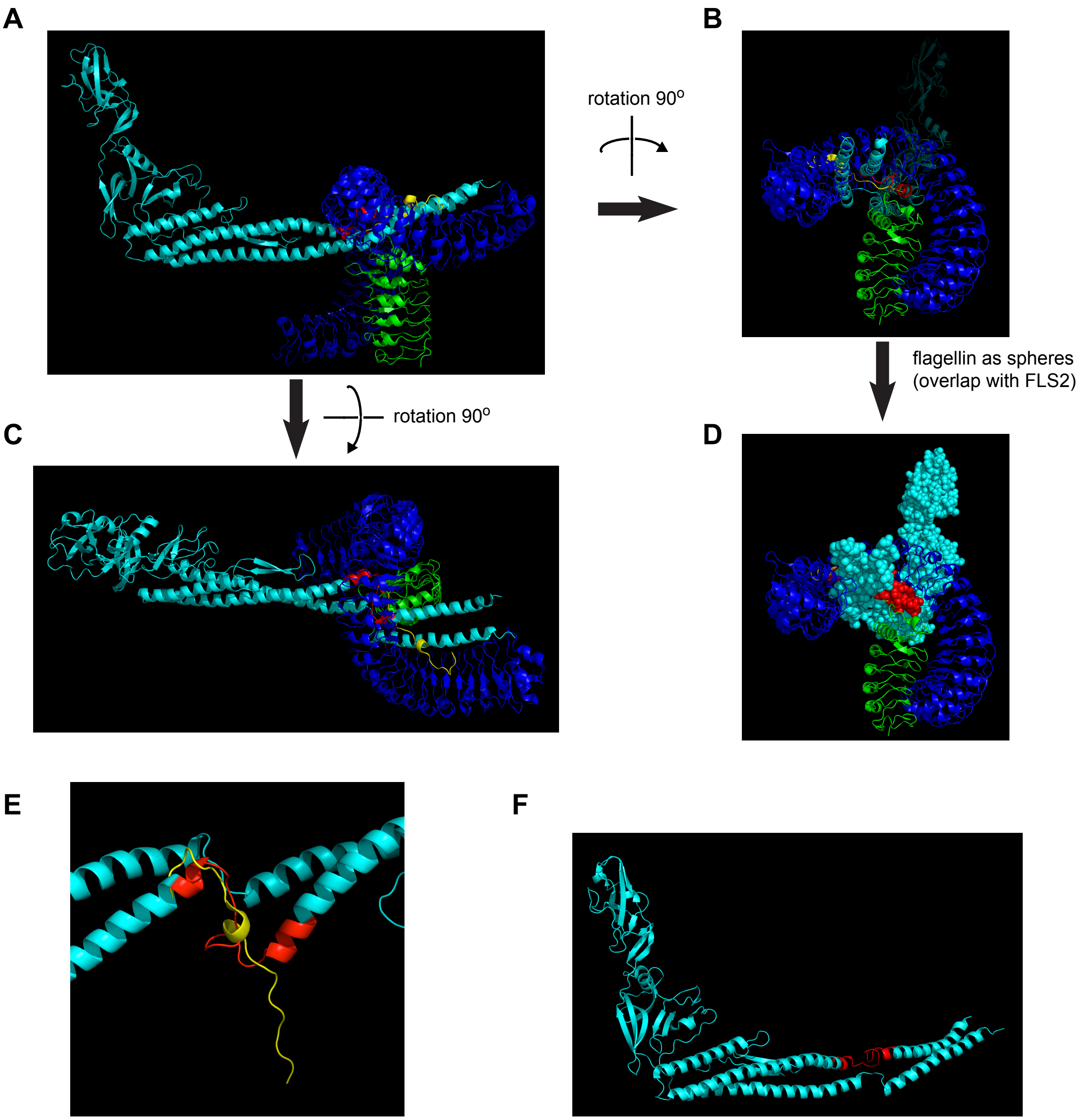

Supplemental Figure 3: Hypothetical docking of full-length flagellin structure (PDB ID: 3A5X) to FLS2-flg22-BAK1 structure (PDB ID: 4MN8) illustrates minimal space for flagellin inside FLS2 LRR, and constraints to flg22 contact with FLS2 LRRs #3-15 if flg22 region is held within full-length flagellin. (A), (B), (C) Flagellin, hypothetically positioned so that flg22 residues within full-length flagellin are near the flg22 binding sites of FLS2 and BAK1. PDB structures 3A5X and 4MN8 superimposed at same scale; (B) and (C) are 90° rotated views of (A). Light blue: flagellin; red: flg22 residues within flagellin (3A5X). Dark blue: FLS2 LRR; green: BAK1 LRR; yellow: flg22 co-crystallized with FLS2 and BAK1 LRRs (4MN8). **(D)** Same view as (C), with space-filling representation of flagellin to more clearly illustrate impossible overlap of flagellin and FLS2 residues in same spatial locations in this arrangement (and other arrangements) of 3A5X and 4MN8. FLS2 and BAK1 side-chains omitted for clarity. **(E)** PyMol alignment of flg22 (yellow, in structure 4MN8) and flg22 region within flagellin (red, in structure 3A5X). Lower portions of flg22 in (E) (the yellow residues that are not proximal to red residues) are the N-terminal 7 residues of flg22 that associate with FLS2 LRRs #3-7 (FLS2 and BAK1 not shown, for clarity). **(F)** Full length flagellin (PDB structure 3A5X) colored as in (A) but shown by itself, showing that flg22 region forms a less-ordered hinge region between flanking pairs of alpha-helical bundles.
